# Evaluating the impact and cost-effectiveness of chlamydia management strategies in Hong Kong: A modeling study

**DOI:** 10.3389/fpubh.2022.932096

**Published:** 2022-07-27

**Authors:** Sandra Montes-Olivas, Yaz Ozten, Martin Homer, Katy Turner, Christopher K. Fairley, Jane S. Hocking, Desiree Tse, Nicolas Verschueren van Rees, William C. W. Wong, Jason J. Ong

**Affiliations:** ^1^Department of Engineering Mathematics, University of Bristol, Bristol, United Kingdom; ^2^Bristol Veterinary School, University of Bristol, Bristol, United Kingdom; ^3^Population Health Sciences, University of Bristol Medical School, Bristol, United Kingdom; ^4^Central Clinical School, Faculty of Medicine, Monash University, Melbourne, VIC, Australia; ^5^Alfred Health, Melbourne Sexual Health Center, Melbourne, VIC, Australia; ^6^Centre for Epidemiology and Biostatistics, Melbourne School of Population and Global Health, The University of Melbourne, Melbourne, VIC, Australia; ^7^Department of Family Medicine and Primary Care, School of Clinical Medicine, Li Ka Shing Faculty of Medicine, The University of Hong Kong, Hong Kong, Hong Kong SAR, China; ^8^Physics Department, University of California, Berkeley, Berkeley, CA, United States; ^9^College of Engineering, Mathematics and Physical Sciences, University of Exeter, Exeter, United Kingdom; ^10^Department of Clinical Research, London School of Hygiene and Tropical Medicine, London, United Kingdom

**Keywords:** chlamydia, cost-effectiveness, economic evaluation, dynamic network models, stochastic model

## Abstract

**Objectives:**

To illustrate the epidemiologic and cost-effectiveness impact of shifting the focus from population-based screening toward a targeted management approach for genital chlamydia infection.

**Design:**

Modeling study, implementing an individual-based, stochastic, dynamic network model.

**Setting:**

Hong Kong.

**Population:**

A hypothetical sample network of 10,000 people with a partnership distribution based on Hong Kong's sexually active population of reproductive age (age 18–49 years).

**Interventions:**

In this study, we present several scenarios with different implementations of universal vs. targeted screening (based on partner numbers). We also explored the impact of (1) screening only, (2) screening plus expedited partner therapy, and (3) screening plus partner testing.

**Primary outcome measures:**

Change of chlamydia prevalence before and after implementing the different strategies. The cost-effectiveness analysis reports total direct cost from a health provider perspective, the QALYs gained, and incremental cost-effectiveness ratios (ICER).

**Results:**

In comparing the effects of universal screening only and targeted screening of the high-risk population, the mean prevalence during the 10th year of intervention was 2.75 ± 0.30% and 2.35 ± 0.21%, respectively (compared with 3.24 ± 0.30% and 3.35 ± 0.21% before the interventions, respectively). The addition of contact tracing to the latter targeted screening scenario reduces the mean prevalence during the 10th year of intervention to 1.48 ± 0.13% (compared with 3.31 ± 0.33% at baseline) in the best-case of testing before treatment and maximal contact-tracing effectiveness (40%). Overall, the most effective scenarios were those for which interventions focused on the high-risk population defined by the number of partners, with contact tracing included. The ICER for targeted screening with contact tracing at 20% and 40% efficiency was $4,634 and $7,219 per QALY gained, respectively (10-year time horizon). Expedited partner therapy did not significantly impact overall chlamydia prevalence and caused overtreatment.

**Conclusions:**

Our study suggests that targeted screening with strengthened contact tracing efforts is the most cost-effective strategy to reduce the prevalence of chlamydia in Hong Kong.

## Introduction

*Chlamydia trachomatis* is the most common bacterial sexually transmitted infection (STI) globally (1). Untreated infections can result in long-term reproductive health consequences such as pelvic inflammatory disease (PID), ectopic pregnancy and infertility in women and infection during pregnancy may result in neonatal blindness, pneumonia or death (2, 3). In men, chlamydia can cause urethritis, proctitis and epididymo-orchitis. Although there is some evidence to support screening to reduce the incidence of chlamydia and hence the complications (4, 5), population-based screening of asymptomatic individuals has been questioned. Opportunistic screening of at-risk populations has not effectively reduced the overall prevalence of chlamydia (6, 7).

Recent discussions have shifted the focus from population-based screening toward strengthening patient management (1, 7). Thus, there are recommendations to move toward a targeted approach instead and strengthen methods to control and prevent the disease (7). For example, best practice sexual health guidelines suggest strengthened case management using contact tracing, retesting for reinfection between 3 and 6 months after chlamydia treatment, and expedited partner therapy (1, 8, 9). Thus, there is a need to evaluate how these additional practices may aid in achieving reductions in chlamydia compared with efforts to increase population-based screening. Although the overall *Chlamydia trachomatis* prevalence was low in Hong Kong at 1.4% (95%CI 0.8–2.5%), sexually active young (18–26 years) women had a relatively high prevalence (5.8%, 95%CI 1.7–18.2%) and a unique U-shape disease burden was observed with peaks in younger and older (40–49 years) women (10). There is currently no national chlamydia screening policy in Hong Kong. Since most chlamydia cases are asymptomatic, control of the disease is a significant challenge even though it can be cured with a course of antibiotics. At the same time, poor awareness, stigma and discrimination associated with STIs in Chinese societies such as Hong Kong prevents contact tracing and stops many from seeking testing and treatment (11).

Mathematical models can explore potential control strategies and incorporate the non-linear dynamics of infectious diseases to optimize interventions. Additionally, these models can give an insight into the cost-effectiveness of these strategies. The model used in this research is an individual-based dynamic transmission model, which represents each individual as a set of states that change over time, allowing large-scale behavior to emerge from small-scale processes.

This study aims to use data from Hong Kong in an individual-based model of chlamydia transmission to illustrate the epidemiologic and cost-effectiveness impact of shifting the focus away from population-based screening toward a targeted approach.

## Methods

We adapted an individual-based, stochastic, dynamic network model of a sexually transmitted infection, previously developed and parameterised for gonorrhea among men who have sex with men (MSM) in the UK (12). This model was originally used to examine antimicrobial-resistant gonorrhea strain development, and we adapted it to represent chlamydia transmission dynamics in a Hong Kong context. The main adaptations were modifying the partnership network to allow only heterosexual partnerships, the presence of a single strain of chlamydia (as opposed to two gonorrhea strains in the previous model), and a single drug therapy treatment.

The stochastic, discrete-time Markov model follows the Susceptible-Infected-Susceptible paradigm (SIS), such that individuals are susceptible to infection through interaction with a sexual partner. In this model, individuals correspond to nodes in a network that accounts for sexual partnerships. Every individual can be infected or susceptible to a single strain of chlamydia. Susceptible individuals become infected though contact with infected partners, with a probability β (per contact per day). Following recovery from the illness after treatment or spontaneous natural recovery, an individual becomes susceptible again. A range of different treatment and intervention pathways are considered, described in Section Intervention scenarios below. We have modified the partnership network to generate a bipartite network that only allows heterosexual partnerships. Similar to the MSM model, the adapted model assumes a scale-free network that obeys a power-law degree distribution, but here each sex has a different power-law exponent (α), as reported by Schneeberger et al. (13). In addition, a small external influx of disease (η) has been added to include the possible input from the mobility of nodes outside the network.

The model was completely re-parameterised to account for the different disease epidemiology, and change in modeled population. By updating sexual behavior parameters according to Hong Kong's population, and implementing various interventions consistent with the Hong Kong setting, we used our updated model to understand the impact and cost of screening interventions. The parameters modified to fit the model to our target population are described in the baseline section and their values can be found in Table 1. We considered the local healthcare perspective for costs and benefits associated with disease prevention to evaluate a setting-specific impact and cost-effectiveness analysis.

**Table 1:** Parameter values, and reference, used in the model scenarios presented in this study.

**Parameter**	**Symbol**	**Baseline**	**Scenarios**	**Reference**
Total population size	N	10,000	Maintained	Selected
Power law exponent	α	Females: 2.8 Males: 2.5	Maintained	Fitted using (10, 14) and MATLAB “polyfit”
Transmission probability	β	0.0016 per sexual partner per day	0.0016–0.0020	Fitted to achieve suitable prevalence value
Natural recovery rate (asymptomatic)	R	0.0027 per day	Maintained	(15)
Fraction symptomatic	P_symp_	10%	Maintained	(16)
Symptomatic testing	P_seek_	62%	Maintained	(17)
Screening rate	γ	0	0.00027–0.0012 per day	Assumed
Tracing efficiency	Ψ	0%	2–40%	Assumed
Extra-network CT import probability	η	Males: 9 ×10^−5^ Females: 0 per person per day	Maintained	Assumed
Interval between screening and returning for treatment (Lab delay + Seek delay)	δ_L_ + δ_S_	N/A	13 days	(18)
Time frame for partnerships network update		1 week	Maintained	Selected
Maximum partners during time frame		10	Maintained	Assumed
Initial chlamydia prevalence		3–4%	Maintained	Fitted

### Model structure

The individualized model has been previously published (12), and further technical details are provided in the Supplementary Material (12). The model has dynamic, stochastic sexual partnership dynamics: individuals are explicitly represented as nodes. Each individual is represented by a (time-varying) vector, indicating their infection status, symptoms or lack thereof, whether they require testing/treatment, flags to represent whether they have been traced or screened, and their associated delays. There are two sexes (male and female), and each node has its own assigned degree denoting its associated number of sexual partners per year. An important part of the model is the ability to represent the dynamics of highly connected individuals or “super-spreaders”. A network restriction algorithm is employed to mimic time-varying partnership networks while preserving long-term network structure; see Zienkiewicz et al. (12) for more details.

The model does not incorporate age structure or site-specific infection. It does contain flexible options for treatment-seeking due to symptoms and asymptomatic screening and includes variable delays according to service provision in time to test and time to receive treatment, as well as options for contact tracing. Model parameters are explained in more detail in the Supplementary Material and are summarized in Table 1.

### Sexual partnership network construction

The underlying sexual partnership network is described using a small number of parameters. This has the advantage of reproducibility and is both fast and straightforward to parameterise. Firstly, the cumulative degree distribution, i.e. the number of sexual partners per person over a given timeframe (the network refreshes once a week), is used to calculate the power-law coefficient. If the partnerships reported are discrepant between men and women (e.g., men typically report a higher mean number of partners than women), this must be adjusted to create a balanced partnership structure across the entire population. Once this static cumulative distribution is described, the whole network of contacts is created for the year using an algorithm to join male and female nodes.

The second stage consists of the time-dependent network generation. This requires two additional parameters: the timeframe over which partnerships can be formed or dissolved, and the restricted maximum degree within the selected timeframe. As this timeframe increases, the simulation approaches the static network. As it decreases, it takes longer to run but can incorporate more realistic partnership dynamics. The original MSM model analysis showed that the underlying annual partnership network was well captured over wide ranges of the partnership timeframe and degree restriction parameters (12). For this study, we updated the partnership network once a week.

### Baseline

We informed our choice of scenarios from recent challenges made to previous control strategies and expert knowledge of the Hong Kong health care system (Table 1). The baseline scenario was obtained according to current chlamydia prevalence and treatment statistics in Hong Kong. The population of interest in the present study are those sexually active, age between 18- and 49-years, whom in the case of Hong Kong was approximately 3,195,400 people in 2020, according to the demographic statistics of the census and statistics department (19). However, a network of that size would require a high computational cost. Thus, we compared different sized networks of 10,000, 20,000 and 1% of the population of interest (31,954) and observed a similar partnership distribution at time 0. Hence, a sample size network of 10,000 people was selected to efficiently run the scenarios presented in this study. The percentage of symptomatic patients was estimated from Korenromp, Sudaryo and de Vlas' study (16), who estimated that symptomatic chlamydia is 11% in males and 6% in females. Since the current model does not differentiate between sexes in relation to the proportion of symptomatic infections, we set the baseline of the symptomatic proportion at 10%. Treatment is given to all those who are symptomatic and seek treatment; we assume that only a proportion of the symptomatic population will seek treatment.

We used data from two surveys performed in Hong Kong (10) to estimate the slope parameters for the power-law distribution of the sexual partnership network. The distribution of the number of partners for both males and females estimated from the surveys was fitted using the 'polyfit' function in MATLAB.

This baseline was used on all intervention scenarios, with a burn-in period of 20 years.

### Intervention scenarios

All intervention scenarios started in endemic equilibrium, in which we had a mean prevalence value in the range of 3–4% in a year. The model provided a range of control options. We first give an overview of the different control option scenarios before describing them in full detail below; Table 2 summarizes the strategies and relevant parameter values. The scenarios present different implementations of three main programs: (1) screening only with no contact tracing; (2) screening plus contact tracing, with treatment of all successfully traced contacts; and (3) screening plus contact tracing, with testing before treatment of successfully traced contacts. We also tested the additional impact of re-testing within 3–6 months, and targeting the screening plus contact tracing to a higher risk population (defined here as those reporting multiple sexual partners in one week) for the above programs.

**Table 2:** Summary of scenarios.

**Scenario**	**Programs**				**Variables**			
	**Universal screening**	**Targeted screening**			
	**Only screening**	**Only screening**	**Screening plus partner tracing**		**Fraction symptomatic**	**Follow-up period**	**Screened population proportion in a year**	**Partner trace efficiency**
			**Treatment to all partners**	**Testing partners before treatment**				
**Non-targeted**							
Ai	X				10%	-	≈10%	-
Aii	X				10%	-	≈30%	-
**Targeted: Follow-up testing of patients seeking treatment**							
Bi		X			10%	3 months	-	-
Bii		X				6 months	-	-
Biii		X				12 months	-	-
Biv			X			3 months	-	40%
Bv				X			-	
Ci		X			30%		-	-
Cii			X				-	40%
Ciii				X			-	
**Targeted: Population with two partners or more**							
Di		X			10%	-	≈10%	-
Dii			X			-	-	2%
Diii			X			-	-	10%
Div			X			-	-	20%
Dv			X			-	-	40%
Ei				X		-	-	2%
Eii				X		-	-	10%
Eiii				X		-	-	20%
Eiv				X		-	-	40%

Scenarios A implements non-targeted screening programs. Scenarios B/C target the symptomatic population seeking treatment by performing follow-up testing. Scenarios D/ E target the population with two or more partners.

The screening programme referred to an approach in which a selected population (either the entire modeled population or a selected sub-population) was tested for chlamydia according to a daily screening rate. This type of test was offered randomly to the selected population without further stratification by age or additional risk factors (e.g., sex workers). In the case of the contact tracing programme, all screened patients and those who voluntarily sought treatment were asked for a list of their partners to trace and notify them and provide treatment depending on the scenario provided. These recent contact partners were traced according to a specific tracing efficiency to account for different compliance scenarios. Afterwards, the traced partners would proceed through two routes: the first one successfully traced partners who would receive treatment without a laboratory test to confirm infection. This type of treatment has been recommended to reduce frontline delay times (20). In the second route, all successfully traced partners were tested and received treatment only when a positive result was returned.

#### Universal screening (scenarios A)

In these scenarios, we applied a universal screening with a screening rate that covers around ~10 and ~30% of the sexually active population per year (see Table 2, scenarios Ai and Aii, respectively). This type of screening is offered randomly in the population without considering factors like the number of partners or symptoms.

#### Targeted screening (scenarios B to E)

##### Follow-up testing of patients seeking treatment (scenarios B and C)

Symptomatic patients with chlamydia who sought treatment would have a follow-up test at a certain period after their appointment/treatment. The waiting period was 92 days (~3 months) for most of the scenarios presented in this study. However, 6 months and 1 year of waiting period were also included (see Table 2, scenarios Bii and Biii, respectively). For the scenarios using this definition of targeted screening, we also tested two different fractions of the symptomatic population: a worst-case scenario with only 10% of the symptomatic population (see Table 2, scenarios B) and a more realistic scenario with a fraction of symptomatic population at 30% (see Table 2, scenarios C).

##### Targeting a higher-risk population (scenarios D and E)

In these scenarios, we changed the screened population to aim at those considered a higher risk. The “higher risk” subpopulation was defined as people in the network with two or more partners in the last week. Scenarios D and E (see Table 2) worked similarly to the universal screening, with treatment to all successfully traced partners (scenario D) or testing before treatment (scenario E), but instead focused on the proportion of the network defined as higher risk. The screening rate was modified to obtain a similar proportion of screened patients as the universal screening scenario (~10% per year). This type of targeted screening did not consider if the patient was symptomatic or not; thus, all scenarios were implemented in a network with 10% symptomatic population. We considered a range of partner trace efficiencies (the proportion of the index case's partners that are traced, on average): 2% (see Table 2 Dii, Ei), 10% (see Table 2 Diii, Eii), 20% (see Table 2 Div, Eiii), 40% (see Table 2 Dv, Eiv).

All simulated scenarios presented in Table 2 were applied during 10 simulated years and replicated 100 times.

### Cost-effectiveness analysis

A cost-effectiveness model was constructed, which takes the outputs of the dynamic transmission model, and uses them to estimate the costs and benefits associated with each of the scenarios described above. The individual-based model returns a time-series of each infection, clinic attendance, treatment, contact tracing, and testing event. So, we can calculate the total costs of each event, for any period, given appropriate costs per intervention. We use the direct costs shown in Table 3. We calculate the direct cost of the interventions in each scenario, in a given year of the control period, as the mean difference between the total direct costs in that year and those in the final pre-control year (across the ensemble of simulations for the relevant scenario.

**Table 3:** Estimated average direct costs of modeled infection events.

**Event**	**Cost (HKD)**	**Reference and assumptions**
Clinic consultation (per attendance)	445	(21)
Testing (per chlamydia test)	700	Hong Kong Department of Health
Contact tracing (per index case)	44.5	Assumed to be 10% of the consultation cost
Treatment (per course of antibiotics)	361	Private practice

The cost analysis method implemented is similar to the one used by Turner et al. (22), in which the direct costs associated with testing, clinic attendance, treatment and contact tracing are estimated and complemented with the saved costs of averted follow-on complications of untreated chlamydia infections. We also model the saved costs of averted follow-on complications of untreated chlamydia infections as a result of the interventions in each scenario and the resulting quality adjusted life year (QALY) gained. We considered six sequalae: four in females–pelvic inflammatory disease (PID), chronic pelvic pain (CPP), ectopic pregnancy (EP), and tubal factor infertility (TFI)–and two in males –epididymitis and urethritis. We take a pragmatic approach, as in Adams et al. (23) to calculate the benefits. Time-series of untreated chlamydia infections by gender are output by the dynamic transmission model, from which each follow-on infection has a probability of occurrence p, and associated health state utility value (HSUV) H & duration T, giving a QALY gained (per complication, per untreated chlamydia infection, p(1-H)T. The mean numbers of sequelae are calculated in post-processing; the model dynamics are independent of any follow-on infections. The values of the parameters used to evaluate each complication, and their associated treatment costs in Hong Kong, are given in Table 4. There is no evidence of the willingness to pay threshold for Hong Kong, thus we present our findings (Table 5) according to 1–3 times the GDP per capita, as per WHO guidance.

**Table 4:** Follow-on complication parameters: probability of occurrence (per untreated chlamydia infection), health state utility value (HSUV), duration and direct financial cost.

**Complication**	**Probability** **p**	**HSUV** **H**	**Duration** **T**	**Cost (HKD)**	**Reference and assumptions**
Pelvic inflammatory disease	10%	0.9	11 days	6,000	(24–27)
Chronic pelvic pain	1.8%	0.69	5 years	4,000	(24–26, 28)
Ectopic pregnancy	0.76%	0.79	4 weeks	20,000	(24–26, 28)
Tubal factor infertility	0.998%	0.76	15 years	50,000	(24–26, 28)
Epididymitis	2%	0.92	1 week	4,000	(25, 27, 29)
Urethritis	75%	0.93	1 week	445	(29)

**Table 5:** Results of the cost-effectiveness analysis: costs per QALY gained.

**Scenario**	**Newly incurred direct**		**Net cost/QALY gain**	
	**cost/QALY gain**			
	**Year 1**	**Year 10**	**Year 1**	**Year 10**
**Non-targeted**				
Ai	$512,499	$343,590	$487,348	$318,438
Aii	$596,685	$423,059	$571,533	$397,907
**Targeted: Follow-up testing of patients seeking treatment**				
Bi	–$110,978	$54,265	–$136,130	$29,113
Bii	$55,798	$67,573	$30,646	$42,421
Biii	–$1,978	$19,254	–$27,130	–$5,898
Biv	$40,943	–$87,493	$15,791	–$112,645
Bv	$90,293	$219,212	$65,142	$194,060
Ci	$115,551	$125,699	$90,399	$100,547
Cii	$79,901	$100,692	$54,749	$75,540
Ciii	$354,610	$255,767	$329,458	$230,615
**Targeted: Population with two partners or more**				
Di	$362,043	$185,584	$336,891	$160,432
Dii	$45,335	$49,740	$20,183	$24,588
Diii	$29,622	$31,921	$4,470	$6,769
Div	$28,511	$30,398	$3,359	$5,246
Dv	$28,845	$30,612	$3,693	$5,460
Ei	$356,063	$200,862	$330,911	$175,710
Eii	$521,497	$322,297	$496,345	$297,145
Eiii	$593,423	$422,323	$568,271	$397,171
Eiv	$634,844	$540,549	$609,692	$515,397

For each scenario (labels as defined in Table 2), the newly incurred direct costs in HKD per QALY gained are shown in columns 2–3 (years 1 and 10 of the control period respectively), and the net change in costs in HKD per QALY gained are shown in columns 3–4 (years 1 10 of the control period respectively). The color scale is from 0 (green) to 3 (red) times HK GDP per capita (HK$357,076 in 2020) (30); negative results are uncolored. Direct costs (columns 2–3) include clinic attendance, treatment, tracing and testing costs as appropriate for each scenario. Net costs (columns 3–4) additionally include the (reduction in) costs due to averted complications; see the text for details.

## Results

**Figure 1:**
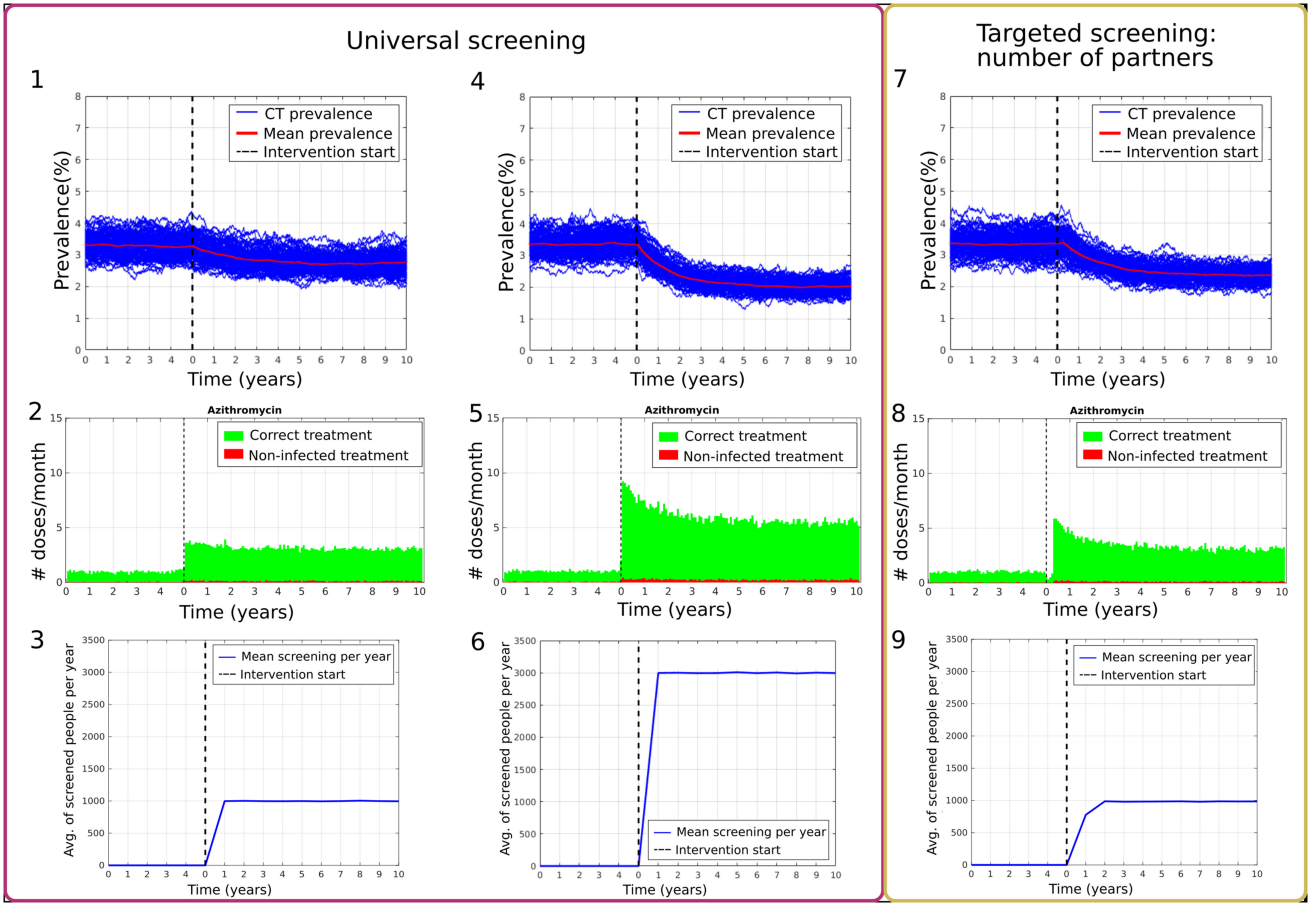
Comparison of program effects between universal screening with 10% and 30% of the population screened per year (**1–3** and **4–6** respectively) and targeted screening according to the number of partners with 10% of the population screened per year **(7–9)**. **(1,4,7)** show prevalence results over 100 simulations (blue) and the mean percentage of prevalence (red). **(2,5,8)** present t the number of doses provided per month, where the individual had a chlamydia infection (green) or was not infected (red). **(3,6,9)** show the average number of people screened per day.

Figure 1 compares the effect of universal screening, scenarios Ai and Aii (Figures 1.1–3 and 1.4–6 respectively) and targeted screening of high-risk population defined by the number of partners, scenario Di (Figures 1.7–9). The change in chlamydia prevalence before and after implementing the two approaches is displayed in Figures 1.1,4,7. The change in the number of treatments provided per month is presented in Figures 1.2,5,8. The main difference between the effect of both approaches shown here is that the targeted approach can reach a higher number of infected individuals at the beginning when reaching a similar number of screenings per year. This means that more treatment is provided during the first months, further decreasing the prevalence before reaching a steady state again. Here we observe that, even when the average number of people being screened a year was similar (see Figures 1.3,9), the prevalence could be reduced further by targeting a specific higher-risk subpopulation of the network. During the last year of the burn-in period, the mean prevalence for scenario Ai (10% universal screening) was 3.24 ± 0.31%, and 2.75 ± 0.30% during the 10th year of the intervention. For scenario Aii (30% universal screening), the mean prevalence was 3.35 ± 0.31% at the end of the burn-in period and 2.01 ± 0.20% during the 10th year of the intervention. On the other hand, the mean prevalence of the burn-in period for scenario Di (targeted screening: 2 partners or more, 10% of the population screened per year) was 3.35 ± 0.38% during its last year and decreased to 2.35 ± 0.21% during the 10th year of intervention.

**Figure 2:**
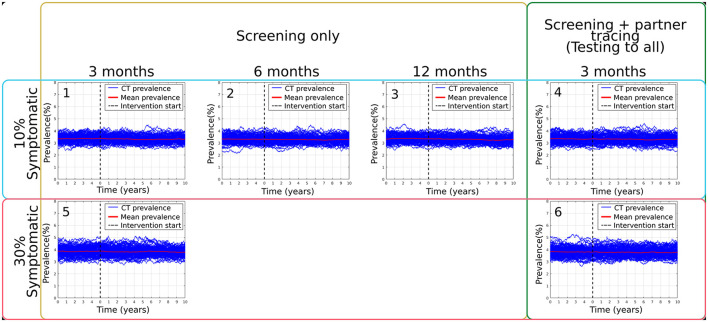
Summary of main results obtained from targeted: follow-up screening simulations, varying follow-up waiting periods, scenarios Bi **(1)**, Bii **(2)**, Biii **(3)**, the modeled percentage of symptomatic population present in the model, as well as the addition of one version of partner tracing on scenarios Biv **(4)**, Ci **(5)** and Cii **(6)**.

The targeted scenarios in which there was a follow-up screening for those patients' seeking attention and all their combinations were also analyzed. However, as shown in Figure 2, we did not observe a significant change in the overall prevalence due to the limited population who presented with symptoms and sought attention. It is important to highlight that this targeted intervention was analyzed by increasing the percentage of the population presenting with symptoms from 10% (Figures 2.1–4) to 30% (Figures 2.5,6) and by changing the follow-up periods on the population of 3 months (Figure 2.1), six months Figure 2.2) and a year (Figure 2.3).

**Figure 3:**
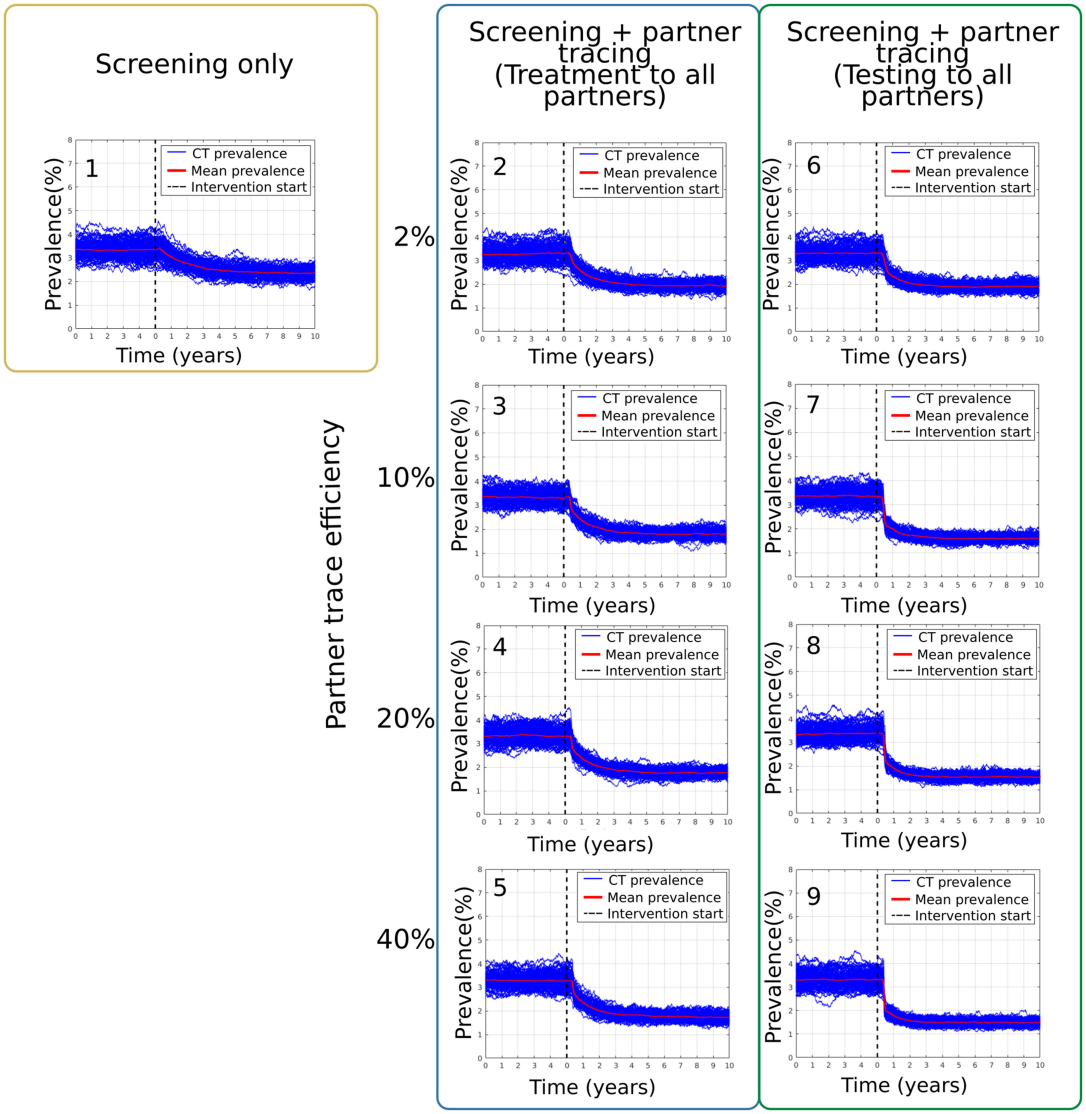
Results from the scenarios targeting the high-risk population as defined by the number of partners. Section **(1)** corresponds to scenario Di. Sections **(2–5)** present the prevalence results from scenarios Dii-Dv and **(6–9)** are from scenarios Ei-Eiv.

On the other hand, the targeted scenarios in which the intervention was focused on the higher-risk population defined by the number of partners provided a more significant reduction of the mean chlamydia prevalence in the sample network. Figure 3 summarizes the main results obtained from the combination of these interventions. For scenario Di, in which screening only was applied as an intervention, the mean prevalence at equilibrium was 3.35 ± 0.38% (see Figure 3.1). After the intervention, this mean prevalence was reduced to 2.35 ± 0.21%. For the best-case scenario of screening plus contact tracing with an effectiveness of 40%, Dv, the mean prevalence changed from 3.27 ± 0.34% to 1.73 ± 0.17% (see Figure 3.5). For comparison, scenario Eiv, which is similar but without over-treating all the traced partners, has a mean prevalence of 3.31 ± 0.33% that was reduced to 1.48 ± 0.13% after the intervention period (see Figure 3.9).

Table 5 reports the results of the cost-effectiveness analysis. Two sets of cost/benefit results are shown for each intervention scenario, based solely on the costs of additional chlamydia treatment, testing, and tracing (columns 2–3) or also including the (saved) costs of averted complications (columns 4–5). The color scale in Table 5 indicates the ratio of the cost per QALY gained to the HK GDP per capita (in 2020), from 0 (green) to 3 (red), for ease of identification of good (green) to poor (red) cost-effectiveness. Note that some cost-effectiveness results returned a negative value (and are not color-scaled), particularly in the case of targeted scenarios in which there was a follow-up screening for those patients seeking attention (Bi, Biii and Biv). These results are caused by the negligible change in incidence (see Figure 2) and consequent negligible change in QALY gain; the effect is that the cost-benefit ratio in these cases is particularly sensitive to small statistical variation (as the ratio of two near-zero quantities) and should be viewed accordingly. Results are shown for years 1 and 10 of the control period, indicating the annual cost-effectiveness in the transient and steady-state, respectively.

In broad terms, the results in Table 5 suggest that the most cost-effective scenarios are those for which the intervention was focused on the higher-risk population defined by the number of partners, with contact tracing included (Dii-Dv). Reasonable-to-good cost-effectiveness can also be obtained in some targeted scenarios in which there was a follow-up screening for those patients seeking attention (Bii, Cii). However, the results in this case are vulnerable to the ratio of small values issue described above and should be interpreted with caution. There is an important cost that is not included in these results, which must be included in any future decision-making. In scenarios where there is contact tracing with treatment to all such traced partners (Biv, Cii, and Dii-Dv), there is extensive over-treatment (i.e., treatment of un-infected traced individuals)–see, for example, Supplementary Figure S8–which would potentially be a driver of antimicrobial resistance. Scenarios where testing precedes treatment for traced individuals (Bv, Cii, and Ei-Eiv) almost eliminate the over-treatment problem but are less cost-effective due to the high testing costs.

Table 6 summarizes the cost-benefit results in an incremental cost-effectiveness ratio (ICER) league table. The process by which scenarios are selected to be reported in the table follows the methodology of Paulden (31) scenarios are ranked by cost and removed if they are “dominated” (have higher cost and smaller or equal benefits than at least one other scenario) or “extendedly dominated” (similarly for a combination of two other scenarios). The results are shown for year 1 of the control period and years 1 to 10, the latter both with and without discounting. The discounting analysis considered both costs and benefits to be subject to discounting, at the same rate of 5%, over the 10-year time horizon of the scenario analysis period, starting at the beginning of the control period. In all cases, three scenarios remain. In order of increasing cost-effectiveness, these are scenarios Biii (follow-up testing of patients seeking treatment after 12 months), Div and Dv (targeted screening of population with two partners or more, with contact tracing at 20 and 40% efficiency, respectively). The corresponding ICERs for scenarios Div and Dv are $3,369 and $6,577 (in year 1), $4,634 and $7,219 (across years 1–10 without discounting), and $4,534 and $7,160 (across years 1–10 with discounting). Other plausible choices of discounting rates lead to the same three scenarios with broadly similar ICER values.

**Table 6:** Results of the cost-effectiveness analysis: ICER league table.

**Scenario**	**Net cost**	**QALY gain**	**Incremental cost**	**Incremental QALY**	**ICER**
Year 1
Only screening with a 12 months follow-up (Biii)	–$884	0.03	-	-	-
Targeted screening with 20% contact tracing efficiency (Div)	$333,661	99.32	$334,545	99.29	$3,369
Targeted screening with 40% contact tracing efficiency (Dv)	$409,345	110.83	$75,683	11.51	$6,577
Years 1–10 total					
Only screening with a 12 months follow-up (Biii)	-$5,708	1.72	-	-	-
Targeted screening with 20% contact tracing efficiency (Div)	$4,120,737	892.16	$4,126,446	890.44	$4,634
Targeted screening with 40% contact tracing efficiency (Dv)	$4,807,866	987.34	$687,129	95.18	$7,219
Years 1–10 total, with discounting of costs and benefits					
Only screening with a 12 months follow-up (Biii)	-$3,949	1.29	-	-	-
Targeted screening with 20% contact tracing efficiency (Div)	$3,265,235	722.27	$3,269,184	720.98	$4,534
Targeted screening with 40% contact tracing efficiency (Dv)	$3,822,986	800.17	$557,751	77.90	$7,160

The table shows all non-dominated and non-extendedly-dominated scenarios, reporting the total net cost and incremental costs (in HKD) and QALY gains, and the ICER (incremental cost/QALY). The results are shown for year 1 of the control period, and for the total of all years 1 to 10 of the control period, the latter both with and without discounting with a discount rate of 5%.

## Discussion

This modeling study evaluates the epidemiologic and economic impact of chlamydia screening approaches in Hong Kong. We add to the literature by providing further evidence for the value to shift resources from a population-based universal screening toward more targeted testing with strengthened patient management. This is consistent with recent calls to rethink strategies to control chlamydia globally (1, 7).

Despite the potential for universal screening to reduce stigma because screening is offered to all irrespective of symptoms or risk behavior, our model showed this was not cost-effective compared to targeted testing with strengthened patient management. Universal screening (which may include opportunistic testing) is recommended in several countries (32–34) but is challenging to scale up and sustain without heavy investments and may not be effective in reducing chlamydia prevalence at the population level. For example, in a cluster-randomized controlled trial in Australia, increasing screening rates from 8 to 20% among 93,828 men and women aged 16 to 29 years had no significant impact on chlamydia prevalence in the community (35).

Our study demonstrated that targeting chlamydia testing toward high-risk individuals, as defined by those with multiple partners, was more cost-effective than universal screening for controlling chlamydia in Hong Kong. Practically, this could be challenging as people may not accurately disclose their sexual activity or under-report the number of sexual partners due to fear or stigma and discrimination. In addition, individuals with multiple partners may be less likely to present to traditional health facilities. There is an urgent need to understand the values and preferences for chlamydia testing and management among high-risk individuals (36). These preference data are helpful to better target individuals with multiple partners to improve the efficiency and cost-effectiveness of chlamydia testing programs. Spurred on by learnings from the COVID pandemic, implementation of novel models of service delivery should be considered, such as improving access points for testing such as point-of-care diagnostics (37), self-testing (38), telehealth or online testing services (39), express STI clinical services (40), and testing in non-clinical settings (41). Concurrently, measures to decrease stigma such as normalizing sexual health checks and routinising sexual health history taking among health providers to better identify those who would benefit from screening the most will be critical for improving the uptake of chlamydia testing among these higher-risk individuals.

Contact tracing of the partners of infected individuals is a cornerstone for STI control. Contact tracing can be patient referral or provider referral (11). Comparing screening only interventions and screening plus contact tracing interventions, we found that the combination of these interventions performs better in reducing the prevalence (and becomes more cost-effective) as the partner tracing effectiveness increases. Prompt evaluation and treatment of sexual contacts are important to interrupt transmission, prevent reinfection of the index case and prevent sequelae from STIs. However, contact tracing has been challenging globally due to the stigma associated with STIs, feeling uncomfortable disclosing an STI diagnosis to sexual partners, fear of relationship breakup, and violence. For example, a qualitative survey from Hong Kong exemplifies the complex struggles of contact tracing for chlamydia (42). There are best practice guidelines to overcome some of these barriers using anonymous notification via SMS or email, assisted by the provider with counseling or contact tracing officers (43).

Providing extra antibiotics or prescriptions for sexual partner(s) of heterosexuals may prevent reinfection of chlamydia in the index patient (8). However, our model demonstrated that expedited partner therapy did not significantly impact overall chlamydia prevalence and caused overtreatment. We showed that partners should ideally be tested before treatment to avoid overtreatment and the potential to worsen antimicrobial resistance. This is particularly important with macrolide use causing a selection pressure on *Mycoplasma genitalium*, syphilis, and Shigella (8, 44, 45). In addition, we found that scenarios that included contact tracing with treatment for all had a slower prevalence reduction in time than those that included screening to all those traced. This could be due to the ability of the model to detect positive patients among those traced who subsequently had their own list of partners to be tested as well. Thus, this allowed for faster detection of infected individuals within sexual networks. Like expedited partner therapy, retesting infected individuals may have benefits at the individual level to detect reinfection earlier, but the population effect was minimal. Together, we show that neither expedited partner therapy nor retesting infected individuals sooner significantly impacted the overall chlamydia prevalence but could still benefit the index patient.

The main strength of this paper is the use of an individual-based model which could more accurately capture sexual partnership dynamics to explore the epidemiologic and cost-effectiveness impact of universal screening compared to strengthening partner management via contact tracing, expedited partner therapy and re-testing for re-infection. We used the latest available data from Hong Kong to contextualize the model. Our findings should be read in light of several limitations. First, consistent with all economic evaluations, the ranking of cost-effective interventions is context-specific and generalisable only to similar countries to Hong Kong. However, we have made the codes of our model freely available so that others can adapt the model to their country's setting. Second, we did not specifically model other populations at higher risk for chlamydia, e.g., sexual minorities or female sex workers. There is uncertainty in Hong Kong regarding whether there is a significant bridging of chlamydia transmission between subpopulations, e.g., between heterosexuals and men who have sex with men. Our model allowed for the seeding of new infections that may account for this potential bridging effect or importation of infections outside the modeled heterosexual population. Similarly, the model does not explicitly account for variations in sexual activity and mixing across different age groups and is parametrised only to population-level data. Last, all models are a simplification of reality, so our findings must be verified in larger population-based studies where possible.

In conclusion, we found that targeted screening with strengthened contact tracing efforts is more effective and cost-effective than universal screening to reduce the prevalence of chlamydia in a Hong Kong context.

## Data availability statement

The original contributions presented in the study are included in the article/Supplementary materials, further inquiries can be directed to the corresponding author/s.

## Author contributions

WW, JO, JH, CF, and KT conceived and designed the research and secured the funding. NV integrated the heterosexual network into the model. SM-O and YO designed and applied the model's simulation scenarios and data analysis. MH and JO performed the cost-effective analysis. All authors contributed to manuscript editing and revision, read and approved the submitted version.

## Funding

Financial support for this study was provided by a grant from the Hong Kong Medical Research Fund (18171282). The funding agreement ensured the authors' independence in designing the study, interpreting the data, writing, and publishing the report. JO is supported by an Australian National Health and Medical Research Council (NHMRC) Emerging Leadership Investigator Grant (GNT1193955). CF is supported by an NHMRC Leadership Investigator Grant (GNT1172900). JH is supported by an NHMRC Senior Research Fellowship (GNT1136117).

## Conflict of interest

The authors declare that the research was conducted in the absence of any commercial or financial relationships that could be construed as a potential conflict of interest.

## Publisher's note

All claims expressed in this article are solely those of the authors and do not necessarily represent those of their affiliated organizations, or those of the publisher, the editors and the reviewers. Any product that may be evaluated in this article, or claim that may be made by its manufacturer, is not guaranteed or endorsed by the publisher.
