# Supplementary material for: Evaluating the impact and cost-effectiveness of chlamydia management strategies in Hong Kong: A modeling study
Source: Front Public Health. 2022 Jul 27;10:932096. doi: 10.3389/fpubh.2022.932096 (PMC9363901; doi:10.3389/fpubh.2022.932096)
Supplement: Supplementary file 1 [file Data_Sheet_1.PDF]

Supplementary Material

1 Comparison of different sized networks

Three different sized networks were generated to compare their partner distributions and the largest connected component of the network. Figure S1 shows the results obtained from these networks at time 0 (i.e., with the initial partnerships and infection status). The full network is displayed in Figures S1 A, E and I; female nodes are represented as diamonds, male nodes as stars, and healthy nodes are coloured blue and infected nodes green. The largest connected node of each network is presented in Figures S1 B, F and J.

Additionally, the distributions of the number of partners (as a percentage of the total population) for females are displayed in Figure S1 C, G and K; and for males in Figures S1 D, H and L. In these bar plots, the data obtained from the two surveys (1, 2) is coloured in orange, and the data obtained from the simulation is in blue.

We observe that the partnership distributions are very stable with respect to the overall size of the population, which gives confidence that a network of 10,000 individuals is sufficient to adequately represent the statistics of a significantly larger population.

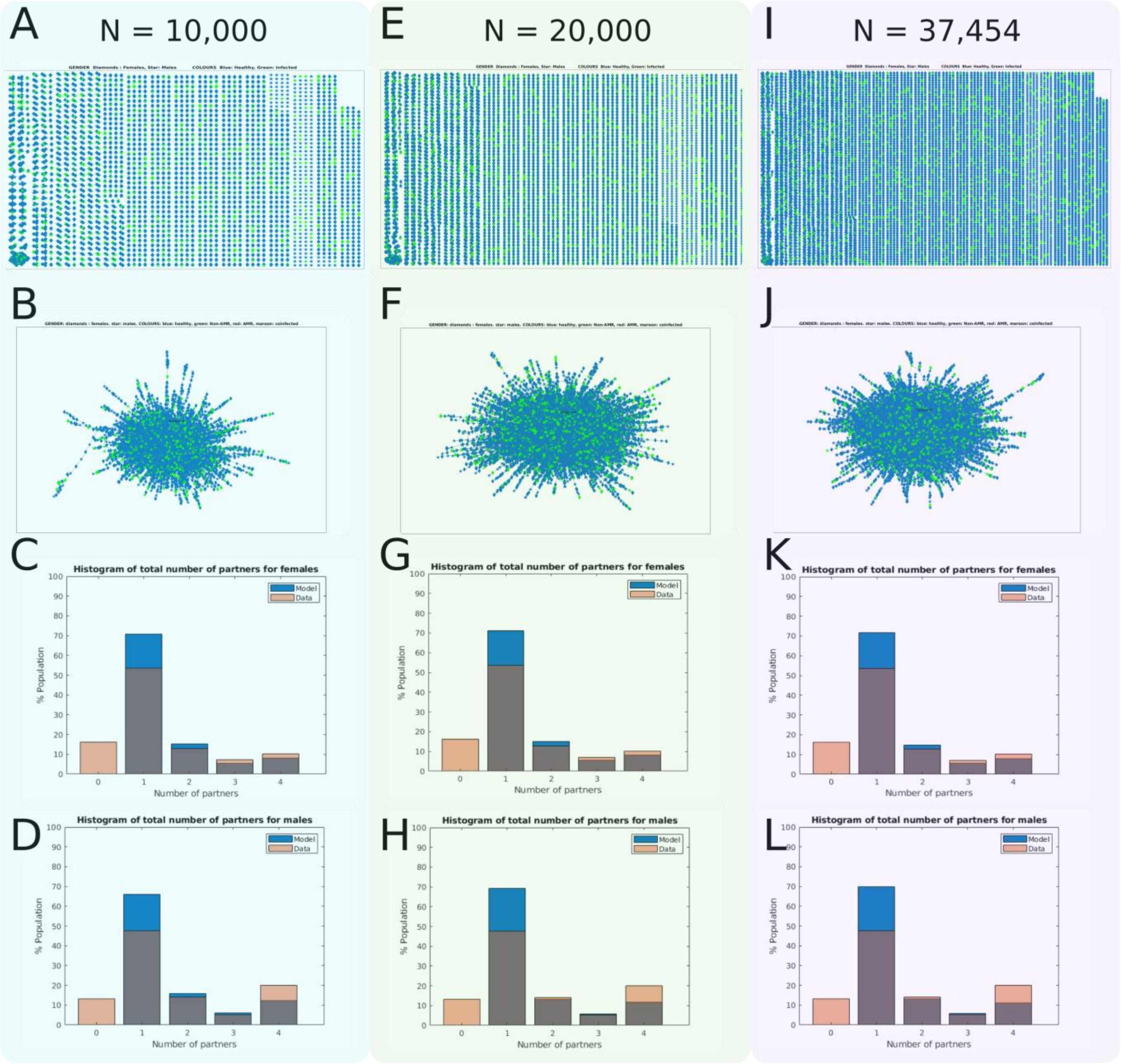

Figure S1. Comparison of three different sized networks at time 0 of the simulation. Sections A-D present results from a network of 10,000 nodes; E-H with 20,000 nodes; and I-L with 37,454 nodes.

2 Follow-up testing of patients seeking treatment

2.1 Variation of the time interval between attendance and screening

Figure S2 shows the effect of changing the waiting period between attendance and re-screening, with a population of 10,000 nodes and fraction symptomatic of 10%; other parameters are as per Table 1 in the main body. The prevalence (Figure S2 A, C and E) and the number of doses of treatment given per month (Figure S2 B, D and F) suggest that there is only a limited effect upon the population, independent of the re-screening period.

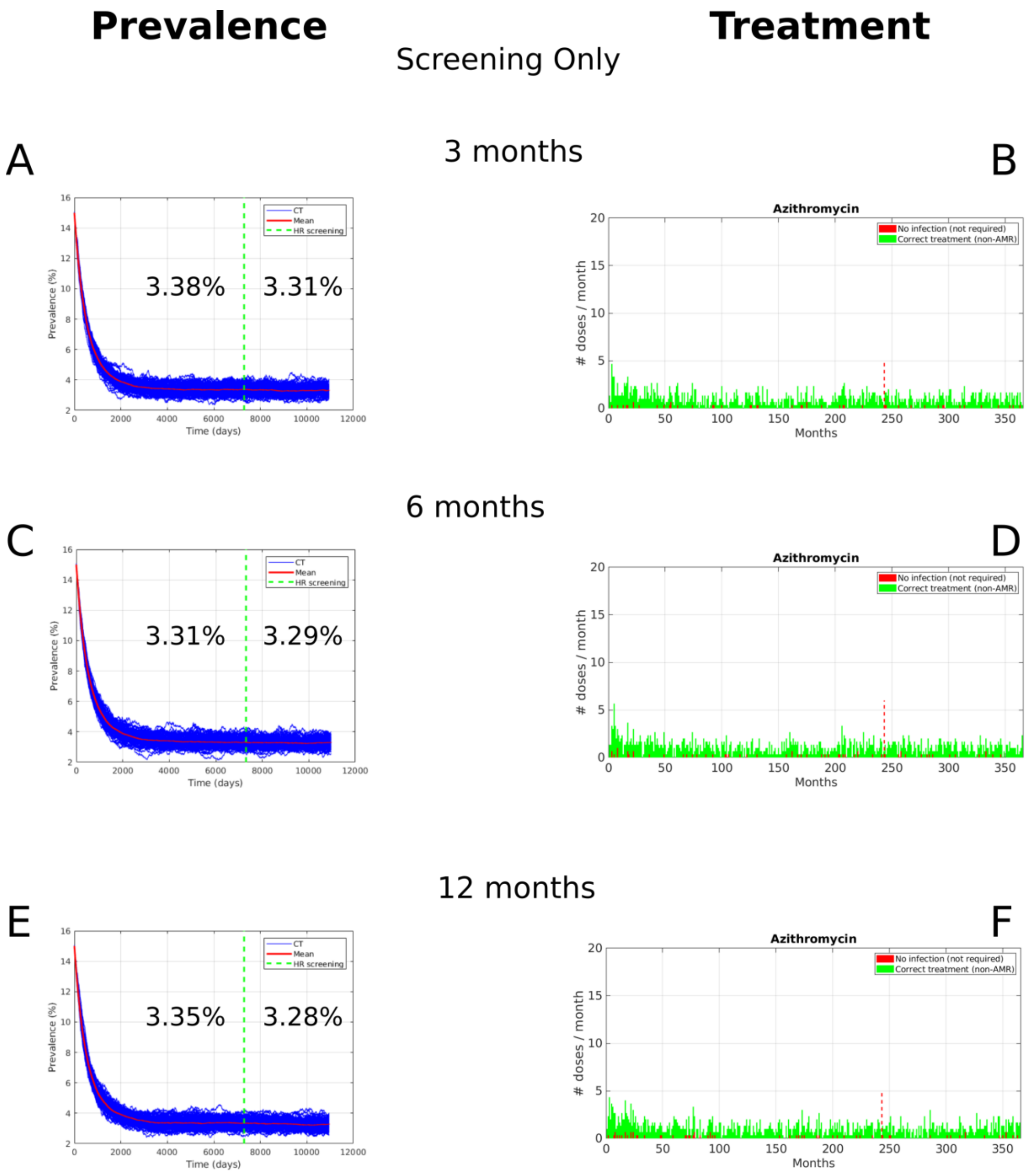

Figure S2. Comparison of different waiting periods for screening after attendance on a targeted approach of symptomatic patients. The chlamydia prevalence is shown in A, C and E for 3, 6 and 12 months waiting periods, respectively. The prevalence percentages are calculated as an average of the last month of the equilibrium period and the last month of the intervention period. The number of the full course of treatments provided per month is presented in B, D and F.

2.2 Simulations with a waiting period of 3 months and a symptomatic fraction of 10%

In the following simulations, we explore the effect of follow-up testing on patients seeking treatment when augmented by partner tracing. A network of 10,000 individuals with a symptomatic fraction of 10% is considered throughout. The prevalence results are shown in Figure S3. We test two different scenarios, where either all traced partners receive treatment (Figure S3.A, C, E and F) or where traced partners are tested before treatment and only receive treatment if they are infected (Figure S3 B, D, F, H), each with four different partner tracing efficiencies: 2% (Figure S3 A, B), 10% (Figure S3 C, D), 20% (Figure S3 E, F) and 40% (Figure S3 G, H). The prevalence values displayed in Figure S3 are calculated as an average of the last month of the equilibrium period and the last month of the intervention period. Similarly, Figure S4 shows the treatment (number of doses per month) results for the same scenarios and tracing efficiencies described above.

The results presented in Figure S4 suggest that the targeted approach of follow-up testing of patients seeking treatment may not be as effective in a population with such a low presence of symptoms. There is no discerning difference between the number of treatments provided during the equilibrium period and those provided after the application of the intervention.

2.3 Simulations with a waiting period of 3 months and a symptomatic fraction of 30%

We also explore follow-up testing of patients seeking treatment when augmented by partner tracing, with the same treatment scenarios and tracing efficiencies as described above, in the case of symptomatic fraction of 30%. All other parameters remain unchanged. The prevalence results are shown in Figure S5, and the treatment doses are shown in Figure S6, with panels as described above.

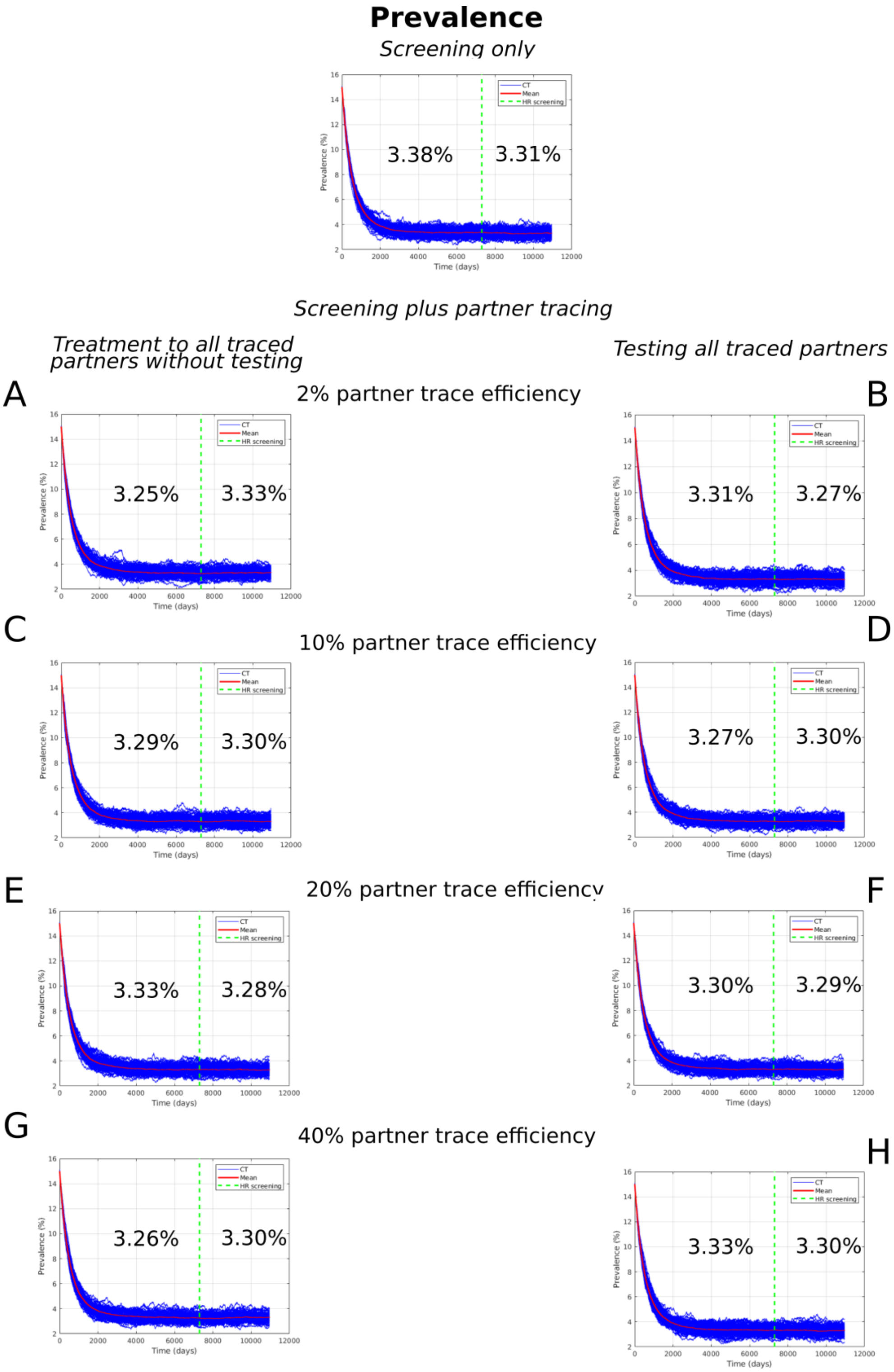

Figure S3. Prevalence comparison of different simulations implementing the targeted screening approach of follow-up patients seeking treatment.

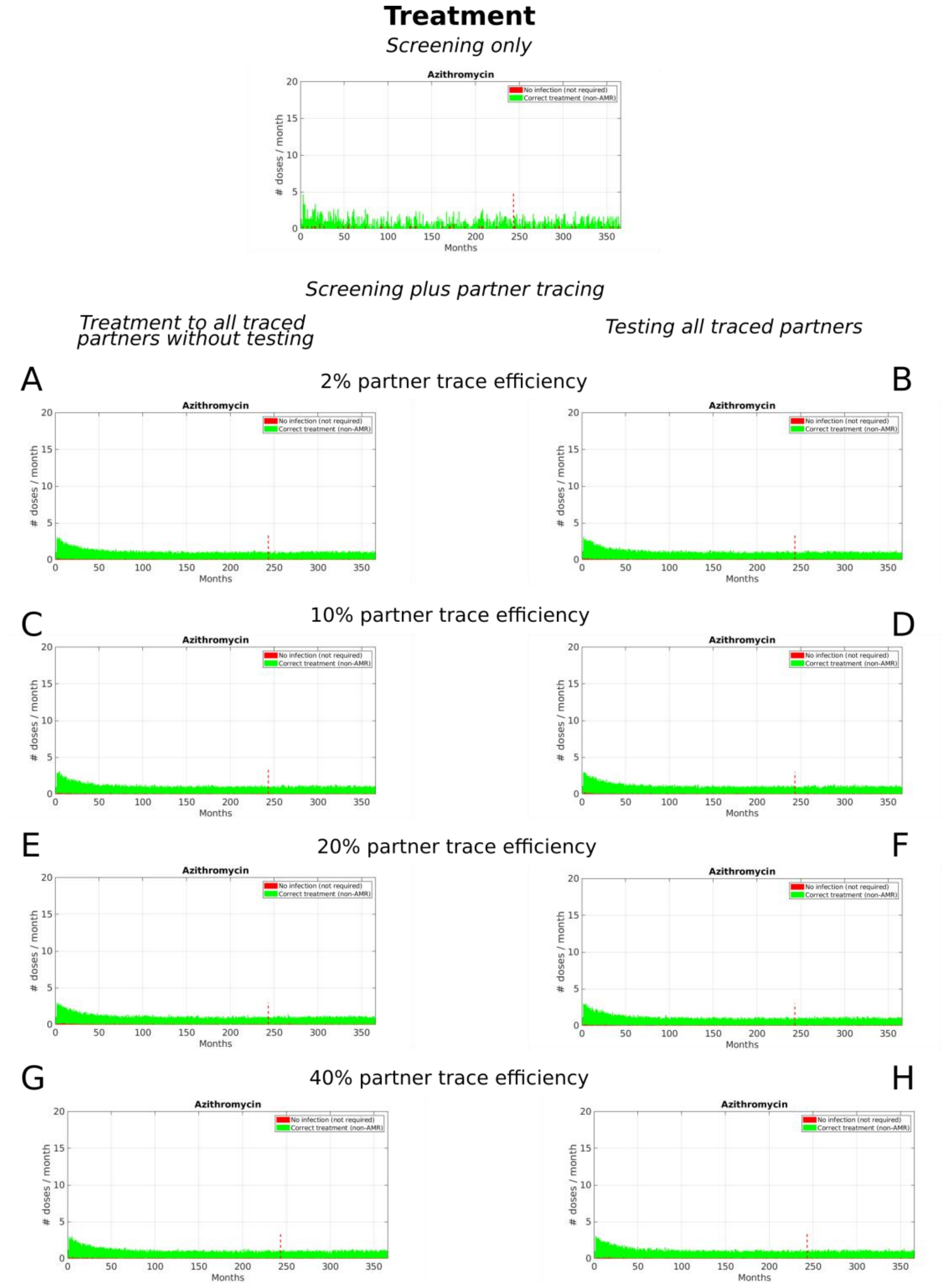

Figure S4. Comparison of the number of treatment doses per month provided before and after the targeted intervention of follow-up screening of seeking patients.

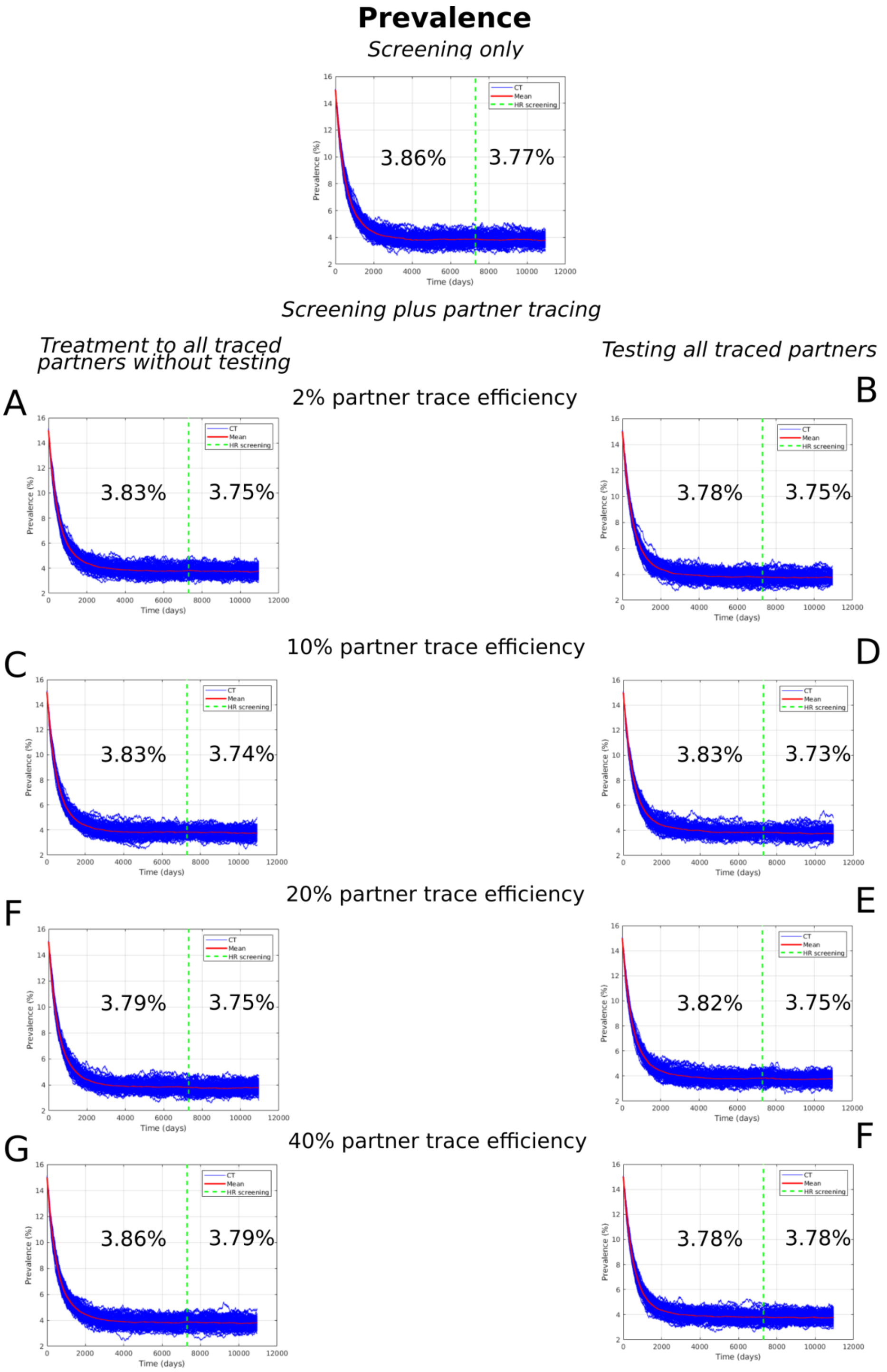

Figure S5. Prevalence comparison of different simulations on a population with a symptomatic fraction of 30%, implementing the targeted screening approach of follow-up patients seeking treatment.

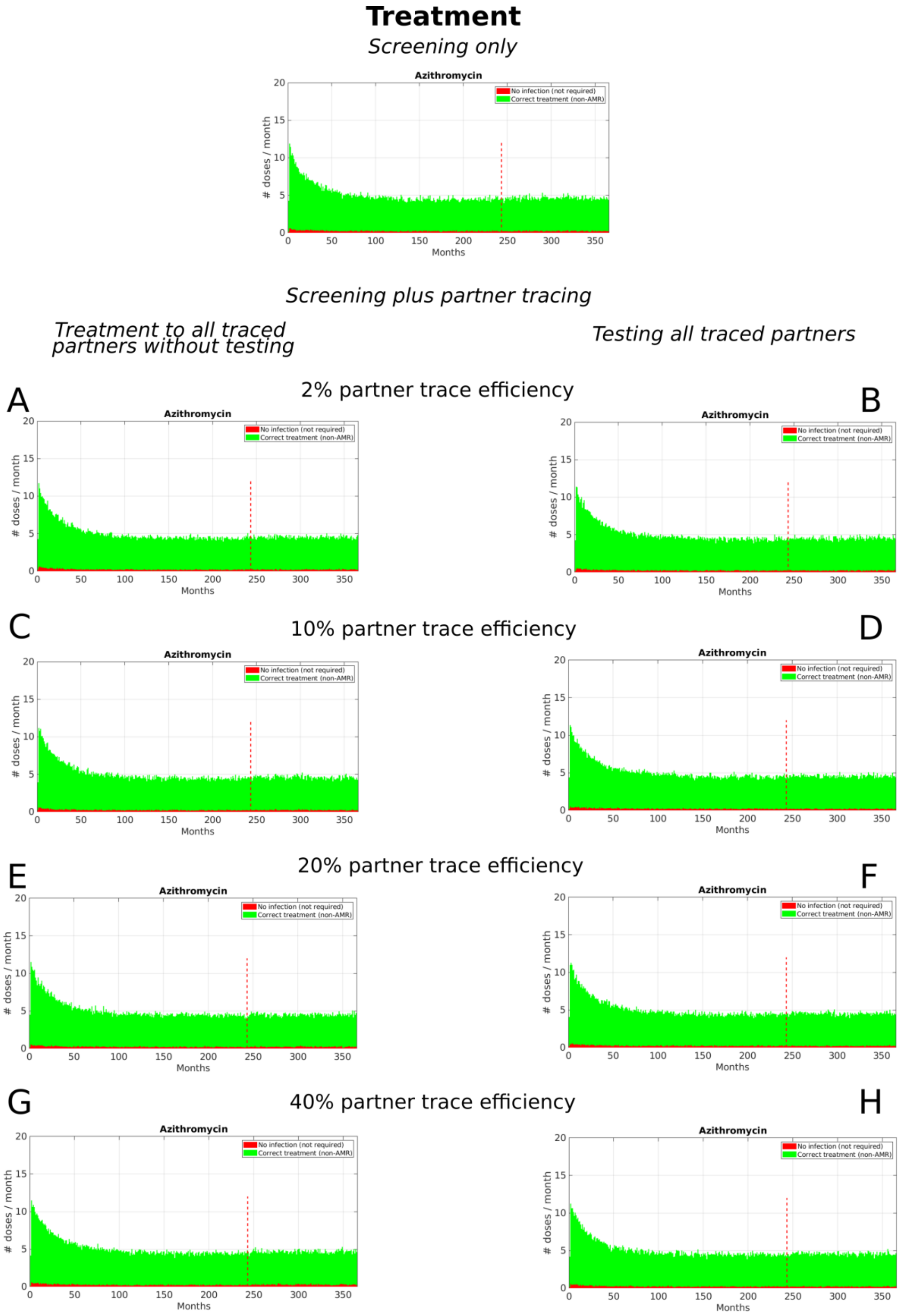

Figure S6. Comparison of the number of treatment doses per month provided before and after the targeted intervention of follow-up screening of seeking patients on a population with a symptomatic fraction of 30%.

2.4 Targeting a higher risk population

Figure S7 compares the mean prevalence in different scenarios of targeting a high-risk population, defined as individuals in the network with two or more partners in the past week. We consider three different scenarios: screening these high-risk individuals only, or additionally tracing their partners and either treating the traced partners irrespective of infection status (Figure S7 A, C, E and G), or testing traced partners before treatment (Figure S7 B, D, F and H). As above, we consider four different tracing efficiencies: 2% (Figure S7 A, B), 10% (Figure S7 C, D), 20% (Figure S7 E, F) and 40% (Figure S7 G, H) The percentages shown in Figure S7 are the mean prevalence obtained during the last month of the equilibrium period (first 20 years of the simulation) and the last month of introducing the targeted screening/tracing (last 10 years of the simulation). Figure S8 presents the corresponding results of prescriptions (dosages per month).

The results suggest that the targeted approach towards screening a high-risk population may be very effective; the prevalence decreases are much larger than in the corresponding scenarios where treatment-seeking individuals act as the index case for treatment or partner tracing. The implication is that targeting high-risk individuals, in terms of the number of sexual partners, can better identify those who are infected but asymptomatic.

The results also show that the scenario where treatment is provided to all those who were traced has a significant number of doses given per month to non-infected individuals. In Figure S8 A, C, E and G, the red section, which corresponds to the treatment given to non-infected individuals, is more significant compared to Figure S8 B, D, F and H, which correspond to the intervention in which all traced partners are tested.

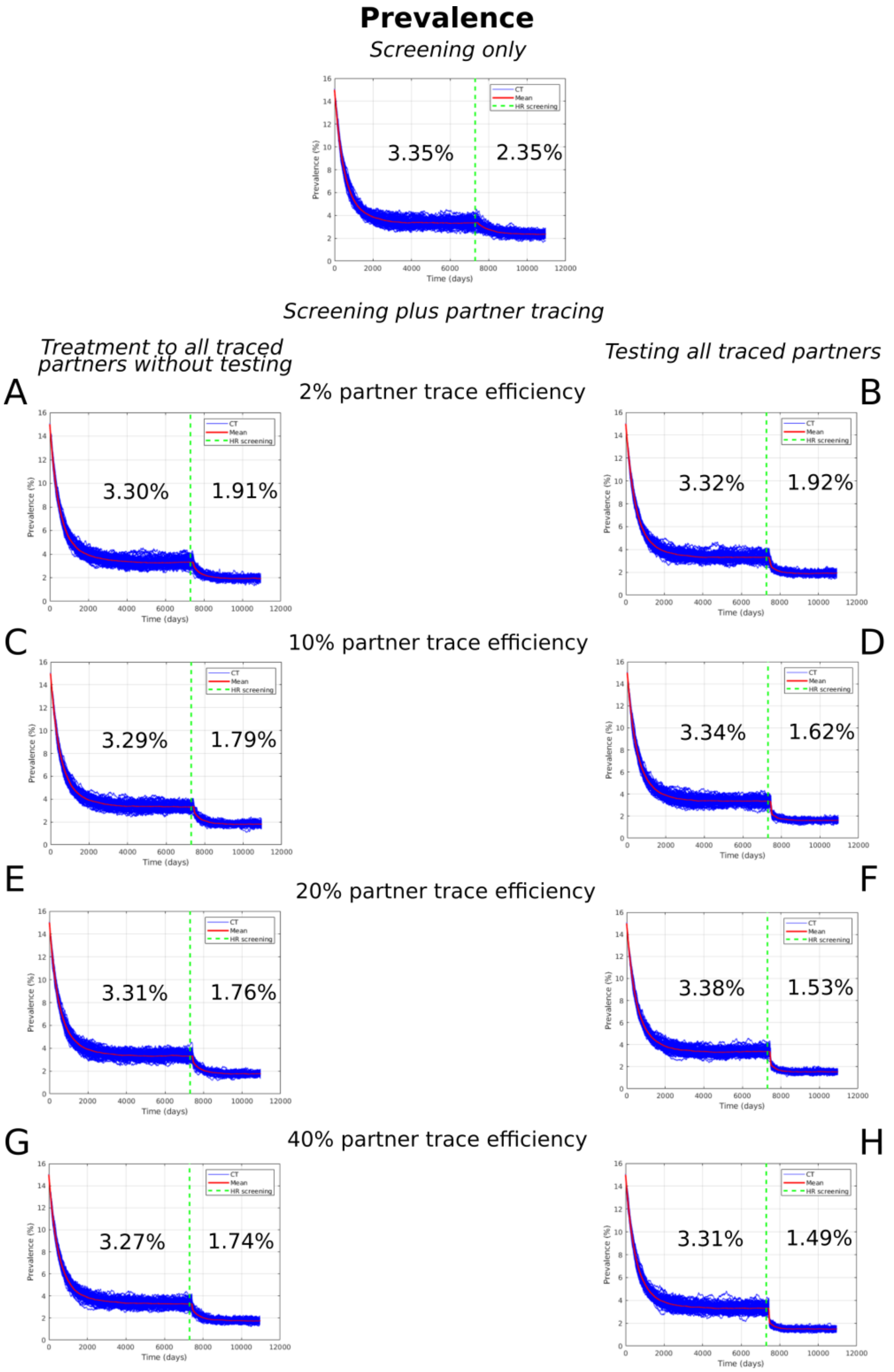

Figure S7. Prevalence comparison of different simulations on a population with a symptomatic fraction of 30%, implementing the targeted screening approach of a high-risk population.

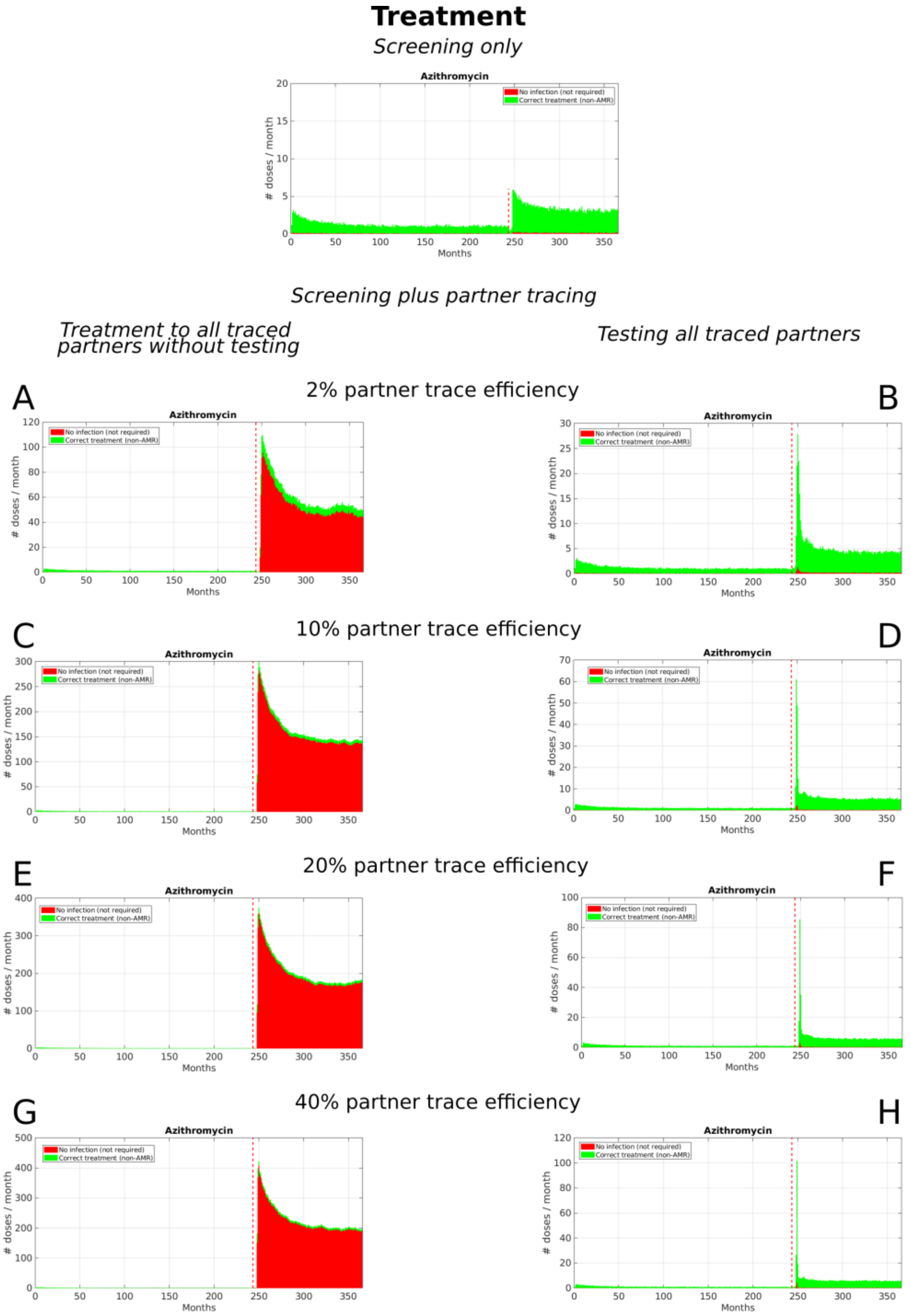

Figure S8. Comparison of the number of treatment doses per month provided before and after the targeted intervention of screening high-risk individuals. The sections in red correspond to doses given to non-infected patients, while the green sections represent those correctly treated. Note the change in scale of the vertical axis due to the significant increase in treatment as tracing efficiency increases.

The model's code is available in the following repository: https://github.com/slmontes/CT_IBM

References:

1. The Family Planning Association of Hong Kong [FPAHK]. The Report of Youth Sexuality Study 2016: The Family Planning Association of Hong Kong; 2017.

2. Wong WC, Zhao Y, Wong NS, Parish WL, Miu HY, Yang LG, et al. Prevalence and risk factors of chlamydia infection in Hong Kong: A population-based geospatial household survey and testing. PLoS One. 2017;12(2):e0172561.
